# Non-Wilms Renal Tumours in Children: The Republic of Ireland Experience

**DOI:** 10.3390/children13040575

**Published:** 2026-04-21

**Authors:** Kris Hughes, Charles Lee, Michael Capra, Jane Pears, Cormac Owens, Michael McDermott, Maureen O’Sullivan, Sri Paran, Israel Fernandez-Pineda

**Affiliations:** 1 Department of Paediatric Surgery, Children’s Health Ireland (CHI) at Crumlin, D12 N512 Dublin, Ireland; hugheskr@tcd.ie (K.H.); charleselee@hotmail.com (C.L.); sri.paran@childrenshealthireland.ie (S.P.); 2 Department of Paediatric Oncology, Children’s Health Ireland (CHI) at Crumlin, D12 N512 Dublin, Ireland; michael.capra@childrenshealthireland.ie (M.C.); jane.pears@childrenshealthireland.ie (J.P.); cormac.owens@childrenshealthireland.ie (C.O.); 3 Department of Pathology, Children’s Health Ireland (CHI) at Crumlin, D12 N512 Dublin, Ireland; michael.mcdermott@childrenshealthireand.ie (M.M.);

**Keywords:** non-wilms renal tumors, pediatric surgery, pediatric renal tumors, clear cell sarcoma of kidney, renal cell carcinoma, congenital mesoblastic nephroma, malignant rhabdoid tumor of the kidney, pediatric oncology, survival outcomes, national cohort study

## Abstract

**Highlights:**

**What are the main findings?**

• Non-Wilms renal tumours (NWRTs) accounted for 17.5% of all paediatric renal tumours nationally in Ireland, with marked heterogeneity in survival by histological subtype (overall survival 76%).
• Outcomes were strongly histology-dependent: 100% survival in congenital mesoblastic nephroma (CMN), clear cell sarcoma of the kidney (CCSK), and anaplastic sarcoma, compared with 43% in renal cell carcinoma (RCC) and 0% in malignant rhabdoid tumour of the kidney (MRTK) with recurrence confined to the RCC subgroup.

**What are the implications of the main findings?**

• These findings reinforce the importance of histology-driven, risk-adapted management, as prognosis and optimal treatment strategies differ substantially between tumour subtypes.
• Persistently poor outcomes in MRTK and relapse risk in paediatric RCC highlight the need for enhanced molecular stratification, targeted therapies, and continued international collaborative research to improve survival in these histological subtypes.

**Abstract:**

**Background**: Non-Wilms renal tumours (NWRTs) are rare paediatric malignancies and account for a small but clinically significant proportion of childhood renal cancers. Due to their low incidence and biological heterogeneity, outcome data are limited, and management is largely extrapolated from international collaborative protocols. No national data describing the incidence and outcomes of NWRTs in children in the Republic of Ireland (ROI) have previously been published. **Objective**: To determine the incidence, treatment strategies, and survival outcomes of NWRTs in children in the ROI. **Methods**: A retrospective cohort study was conducted of all children under 16 years of age with histologically confirmed renal tumours diagnosed and treated at Children’s Health Ireland (CHI) at Crumlin between January 2005 and December 2025. As CHI Crumlin is the single national paediatric oncology centre in the ROI, this cohort represents national case ascertainment for the study period. A total of 143 paediatric renal tumours were identified; Wilms tumours (*n* = 118) were excluded, leaving 25 children (17.48%) with NWRTs for analysis. No cases of bilateral renal tumours were identified. Histological subtypes included renal cell carcinoma (RCC), clear cell sarcoma of the kidney (CCSK), congenital mesoblastic nephroma (CMN), malignant rhabdoid tumour of the kidney (MRTK), and anaplastic sarcoma of the kidney. Demographic characteristics, treatment strategies, and survival outcomes were analysed. **Results**: Twenty-five children with NWRTs were identified: CCSK (*n* = 9), RCC (*n* = 7), CMN (*n* = 6), MRTK (*n* = 2), and anaplastic sarcoma of the kidney (*n* = 1). At a median follow-up of 107.9 months (range 4.5–181.3 months), overall survival for the cohort was 76%. Overall survival by histology was 100% for CMN, CCSK and anaplastic sarcoma, 43% for RCC, and 0% for MRTK. Treatment strategies varied by histology, with 68% undergoing upfront surgery, 32% receiving neoadjuvant chemotherapy, 60% receiving adjuvant systemic therapy, and 44% receiving radiotherapy. Tumour recurrence occurred in 4/25 patients (16%), confined to the RCC (3) and CMN (1) subgroups. Seven Event-Free Survival events were observed, comprising three RCC relapses and one RCC progression, one CMN relapse, and two MRTK progression-related deaths. No recurrences occurred in CCSK. **Conclusions**: NWRTs comprised 17.5% of all paediatric renal tumours diagnosed nationally during the study period and demonstrated marked heterogeneity in outcomes according to histological subtype. CMN showed excellent survival with six out of seven requiring surgery alone, whereas MRTK remained associated with dismal outcomes despite multimodal therapy. These national data support histology-driven, risk-adapted management and highlight the importance of continued international collaboration to improve outcomes in NWRTs.

## 1. Introduction

Paediatric renal tumours are uncommon but clinically significant malignancies requiring multidisciplinary management and age-adapted treatment strategies. Wilms tumour (WT), or nephroblastoma, accounts for approximately 90–95% of malignant paediatric renal tumours in European and North American series, whereas non-Wilms renal tumours (NWRTs) represent a biologically heterogeneous minority associated with distinct molecular drivers, treatment responsiveness and clinical outcomes [1,2,3,4,5]. Several NWRT entities demonstrate limited sensitivity to standard WT chemotherapy, making early diagnostic discrimination and expert pathology review essential to optimise care [2,6].

NWRTs encompass congenital mesoblastic nephroma (CMN), clear cell sarcoma of the kidney (CCSK), malignant rhabdoid tumour of the kidney (MRTK), paediatric renal cell carcinoma (RCC) and rarer subtypes. Advances in molecular diagnostics including ETV6-NTRK3 fusion in cellular CMN, BCOR internal tandem duplication or YWHAE-NUTM2 fusion in CCSK, and SMARCB1 loss in RTK have improved classification and risk stratification, particularly where morphology overlaps with WT [6,7,8,9,10,11].

International population-based studies demonstrate age-standardised incidence rates of paediatric renal tumours of approximately 9.1–9.8 per million person-years in Europe and North America, with malignant NWRTs accounting for roughly 1 per million children annually [3,4,12]. Despite their rarity, NWRTs remain clinically important due to the aggressive biology of selected subtypes. A national cohort from The Netherlands (1990–2014) highlighted differences in outcomes between tumour entities, with WT dominating incidence but RTK associated with substantially poorer survival [5].

Comparable registry analyses from Greece and the US SEER programme report similarly low absolute incidence rates for malignant NWRTs, emphasising the challenges of interpreting subtype-specific trends from single-country datasets [6,12]. These limitations have supported the development of collaborative frameworks and centralised pathology review to improve diagnostic accuracy and facilitate meaningful outcome reporting across Europe.

Hospital-based tertiary-centre cohorts provide complementary insight into treatment pathways and relapse patterns. In a large institutional series from India, NWRTs accounted for 19% of paediatric renal tumours; outcomes varied considerably by tumour subtype, highlighting the biological heterogeneity of this group [13].

Clinical presentation and imaging findings frequently overlap between WT and NWRTs, limiting the reliability of radiology alone for subtype discrimination. Integration of age at presentation, tumour characteristics and early treatment response are therefore essential, with specialist pathology review, percutaneous biopsy and molecular profiling recommended when atypical features are present [2,6].

The SIOP-RTSG UMBRELLA framework has facilitated centralised pathology review and integrated molecular diagnostics across Europe and the Republic of Ireland (ROI), enabling more accurate classification and supporting collaborative research for rare renal tumour subtypes [6]. Treatment approaches vary substantially across NWRT entities: CMN is typically managed with surgery alone, whereas CCSK and RTK require intensive multimodal therapy, and RCC management centres on complete surgical resection with stage-directed systemic treatment [2,6,14,15,16]. Population-based analyses report differing survival outcomes between subtypes, underscoring the importance of tumour biology in guiding management strategies [5].

Within the ROI, publicly available registry data provide broad summaries of childhood cancer incidence but limited detail regarding renal tumour subtypes and outcomes. Given the rarity of NWRTs and the reliance on European cooperative-group infrastructure for diagnosis and management, national-level outcome data are required to contextualise Irish practice within the wider European experience and to support benchmarking against international cohorts [6,17].

Accordingly, this study describes the national experience with NWRTs in children treated at the single national paediatric oncology centre, focusing on incidence, treatment strategies and long-term outcomes within a contemporary European multidisciplinary framework.

## 2. Materials and Methods

### 2.1. Study Design and Setting

A retrospective cohort study was conducted at Children’s Health Ireland (CHI) at Crumlin, the Republic of Ireland’s sole national paediatric oncology centre. All children diagnosed with NWRTs between January 2005 and December 2025 were identified by cross-referencing multiple institutional sources, including the pathology registry, the paediatric oncology database, and surgical records, with further validation through the National Cancer Registry Ireland. This multi-source approach ensured comprehensive national case ascertainment. This study’s administrative census date was 31 December 2025. Patients who had not experienced the primary event (disease relapse) or were lost to follow-up were right censored at either their last known contact or the study end date, whichever occurred first. To ensure a representative cohort, all patients diagnosed up to the end of the study period were included in the analysis.

### 2.2. Patient Selection

Children aged <16 years with histologically confirmed primary NWRTs were included. No patients with non-Wilms bilateral renal tumours were observed in the study period. Only malignant or clinically relevant renal tumours of interest were included; benign tumours were not part of the study cohort. Clinical data completeness was verified for all included patients. The presence or absence of underlying cancer predisposition syndromes was recorded and analysed descriptively.

### 2.3. Histopathological Diagnosis and Classification

All tumour diagnoses were confirmed locally by specialist paediatric pathology review, supported by immunohistochemistry where indicated. Tumour molecular testing was selectively conducted based on specific histological features or clinical suspicion of a genetic predisposition syndrome. Given the extended study period, histological classification reflected contemporary diagnostic standards at the time of treatment, incorporating evolving integration of SIOP, and in earlier years, Children’s Oncology Group (COG) diagnostic frameworks. Molecular diagnostics became routinely used in our institution in 2010. RT-PCR for ETV6:NTRK3 fusion (diagnostic of congenital cellular mesoblastic nephroma) was introduced in 2010. Our pathology research group discovered and described the YWHAE::NUTM2 fusion in CCSK and has been running BCOR ITD assays by PCR as per need since 2018. Full RNA fusion and DNA mutational panels by NGS were introduced in 2022. Molecular testing is done on tumour samples for somatic mutation detection. All cases of rhabdoid tumour and also anaplastic sarcoma of kidney are routinely tested for germline mutation of SMARCB1 and DICER1, respectively. This involves clinical genetics and formal germline testing following detailed informed consent. Oncomine OCCRA DNA and RNA panels are used for NGS testing.

### 2.4. Diagnostic Evaluation and Pre-Operative Strategy

Diagnostic work-up included cross-sectional abdominal imaging with MRI and thoracic staging with CT. Biopsy was performed selectively in accordance with prevailing SIOP-based principles, particularly when imaging or clinical features suggested NRWT histology or when diagnostic uncertainty existed. Patients with typical WT features based on age and imaging were not routinely biopsied prior to initial management.

### 2.5. Surgical Management

Radical nephrectomy with routine regional lymph node sampling was the standard surgical approach. Nephron-sparing surgery was not performed within this cohort. Operative strategy and management of vascular involvement were individualised according to tumour characteristics and multidisciplinary team discussion.

### 2.6. Systemic Therapy and Radiotherapy

Neoadjuvant and adjuvant treatments were determined by tumour histology and stage within a multidisciplinary framework, primarily guided by SIOP-based protocols. Selected patients with RCC received targeted therapy, including tyrosine kinase inhibitors, where clinically indicated. All NWRT histologies were staged and managed in accordance with SIOP-aligned frameworks. Radiotherapy decisions were histology- and risk-adapted.

### 2.7. Follow-Up

Follow-up schedules were tailored to tumour subtype. Patients with CMN typically underwent short-term surveillance following complete resection, whereas children with other NWRTs were followed at approximately three-month intervals during the first two years, six-month thereafter, and annually in longer-term follow-up, consistent with institutional practice.

### 2.8. Outcomes and Definitions

The primary outcome was overall survival (OS), defined as the time from the date of initial diagnosis to the date of death from any cause. Secondary outcomes included Event-Free Survival (EFS), defined as the time from diagnosis to the first occurrence of disease progression, recurrence, secondary malignancy, or death from any cause. Other secondary outcomes included recurrence rate, stage distribution, and treatment-related morbidity. Recurrence was defined as radiological or histologically confirmed tumour relapses following completion of primary therapy.

### 2.9. Statistical Analysis

All descriptive analyses, statistical analyses, and Kaplan–Meier curves for OS and EFS were generated using IBM SPSS Statistics version 30.0.

## 3. Results

### 3.1. Cohort Characteristics

**Figure 1:**
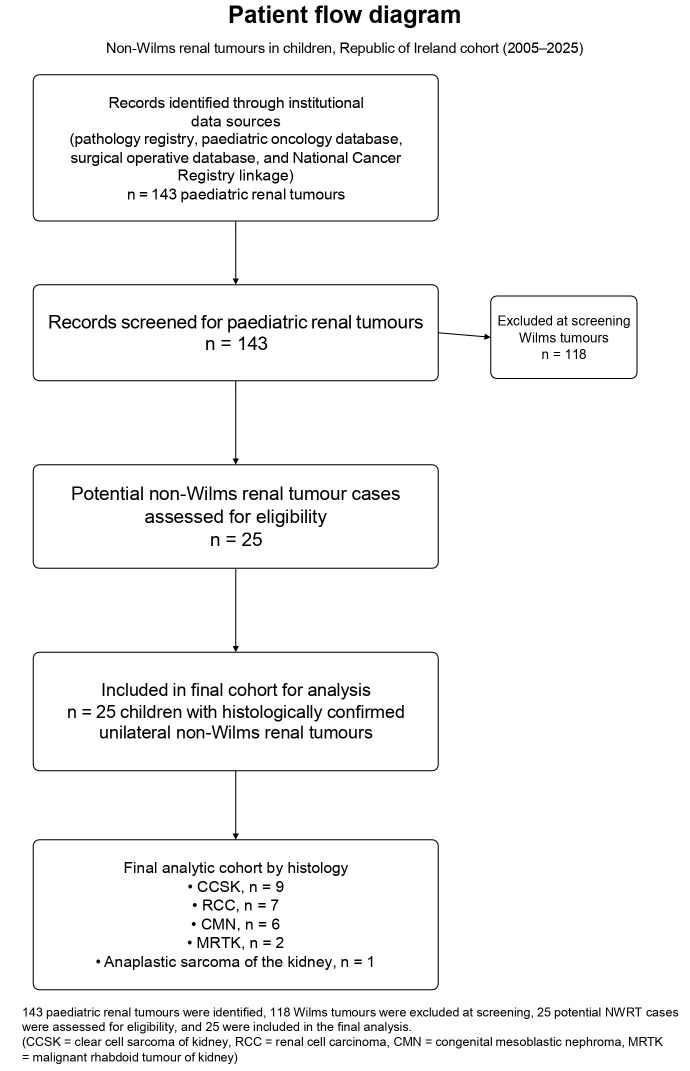
Patient flow diagram.

A total of 25 children (17 females and 8 males) with NWRTs were identified during the study period (patient flow diagram in Figure 1). Histological subtypes included CCSK (*n* = 9), RCC (*n* = 7), CMN (*n* = 6), MRTK (*n* = 2), and anaplastic sarcoma of the kidney (*n* = 1). Baseline clinicopathological characteristics by histology are summarised in Table 1. Three CMN subtypes were identified: classic (1), cellular (4), and mixed (1).

### 3.2. Clinical Presentation and Diagnostic Evaluation

Clinical presentation varied by tumour subtype. Overall, abdominal mass was the most common presenting feature, while haematuria and flank pain were less frequent. Presenting symptoms were documented in 19 of 25 patients.

Among the seven RCC patients, presenting complaints were available for five: three presented with a palpable abdominal mass (one associated with flank pain), one presented with isolated flank pain, and one with haematuria. In the CCSK cohort, presenting symptoms were documented in seven of nine patients and included abdominal mass (*n* = 3), flank pain (*n* = 2), haematuria (*n* = 1) and ptosis (*n* = 1). The single anaplastic renal sarcoma presented with haematuria. Among patients with congenital mesoblastic nephroma (CMN), presenting complaints were documented in five of six patients and included abdominal mass (*n* = 2), antenatal diagnosis (*n* = 2) and haematuria (*n* = 1).

Baseline staging included CT imaging of the thorax, abdomen and pelvis. Additional staging investigations were performed selectively. Within RCC patients, CNS imaging was performed in 4/7 and nuclear medicine bone scan in 5/7, with one patient undergoing PET-CT. Among CCSK patients, nuclear medicine bone scan was performed in 8/9 and CNS imaging in 6/9; one patient did not undergo advanced staging imaging. Both MRTK patients underwent CNS imaging (2/2), with one also receiving a nuclear medicine bone scan (1/2). The anaplastic sarcoma patient underwent both CNS imaging and a nuclear medicine bone scan (1/1).

### 3.3. Disease Stage at Diagnosis

Staging at diagnosis was classified as either localised disease or Stage IV metastatic disease based on radiological findings. Among RCC patients, two presented with Stage IV disease, with one patient presenting with metastases of the upper abdominal wall and another with pulmonary metastases, while four had localised tumours. One RCC patient had a prior diagnosis of right psoas rhabdomyosarcoma with pulmonary metastases and was subsequently found to carry a pathogenic TP53 mutation. A renal mass was later detected during surveillance imaging for the original malignancy.

Within CCSK, one of nine patients presented with Stage IV disease with osseous metastases, while eight had localised disease. The anaplastic sarcoma patient presented with Stage IV disease with bone metastases. All CMN patients presented with localised disease. Both MRTK patients presented with Stage IV disease, with one presenting with metastasis to the CNS and the other having lung metastasis.

### 3.4. Treatment Patterns

Treatment strategies by histology are summarised in Table 2. Overall, 17/25 patients (68%) underwent upfront surgery, 8/25 (32%) received neoadjuvant chemotherapy, 15/25 (60%) received adjuvant systemic therapy, and 11/25 (44%) received radiotherapy, consistent with the treatment distribution shown in Table 2.

Upfront surgery was performed in all CMN and MRTK patients, while neoadjuvant chemotherapy was predominantly administered in CCSK (7/9) with one RCC (1/7) patient receiving neoadjuvant based on an initial diagnosis of WT. Adjuvant chemotherapy was delivered in all CCSK patients, three RCC patients, one CNM patient, the single anaplastic sarcoma patient, and one MRTK patient. Targeted therapies were used exclusively in RCC (4/7).

Pre-treatment biopsy was performed in 5/7 RCC patients. Among CCSK patients, 8/9 underwent biopsy prior to treatment. No CMN patients underwent biopsy, while one MRTK patient underwent biopsy.

### 3.5. Treatment by Histological Subtype

#### 3.5.1. RCC

In the RCC cohort, all six patients underwent upfront surgery with lymph node sampling, with three cases identifying positive nodal involvement. Notably, a misdiagnosed WT case received neoadjuvant chemotherapy, ultimately requiring aggressive multimodal therapy including second-look surgery and palliative immunotherapy following papillary RCC diagnosis, while others received varied adjuvant chemotherapy regimens and targeted therapies.

#### 3.5.2. CCSK

Seven of nine CCSK patients received neoadjuvant chemotherapy, while all underwent adjuvant therapy involving doxorubicin, cyclophosphamide, etoposide, vincristine, and carboplatin. Radiotherapy was delivered to seven patients, primarily targeting the flank, and histological evaluation identified positive lymph nodes in one case.

#### 3.5.3. Anaplastic Sarcoma of the Kidney

The patient with anaplastic sarcoma of the kidney underwent upfront surgery with negative lymph node sampling. Following surgery, treatment consisted of adjuvant multi-agent chemotherapy (actinomycin D, doxorubicin, ifosfamide, and vincristine) and flank radiotherapy at 50.4 Gy.

#### 3.5.4. CMN

All CMN patients underwent upfront surgery, with only one case requiring adjuvant chemotherapy (vincristine and actinomycin D) and no patients receiving radiotherapy. Lymph node sampling, conducted on 50% of the cohort (3/6), showed no evidence of disease.

#### 3.5.5. MRTK

Both MRTK patients underwent upfront surgery and node sampling, which confirmed lymph node involvement. While neither received neoadjuvant treatment, one case was managed with palliative intent, and the other received adjuvant chemotherapy with vincristine, actinomycin D, doxorubicin, and flank radiotherapy (19.8 Gy).

### 3.6. Survival Outcomes

**Figure 2:**
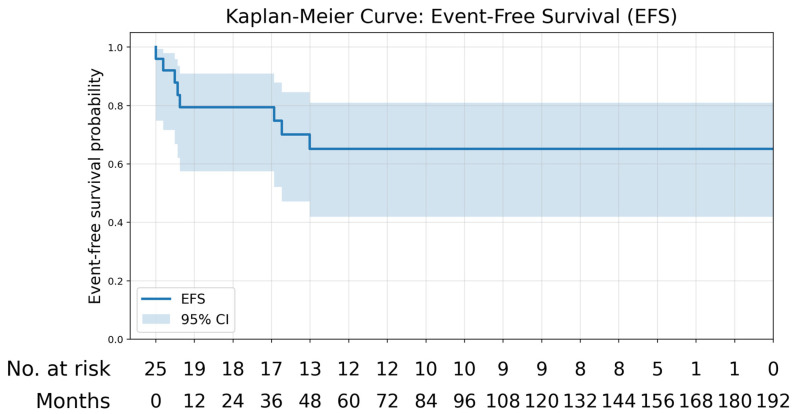
Kaplan–Meier Event-Free Survival.

**Figure 3:**
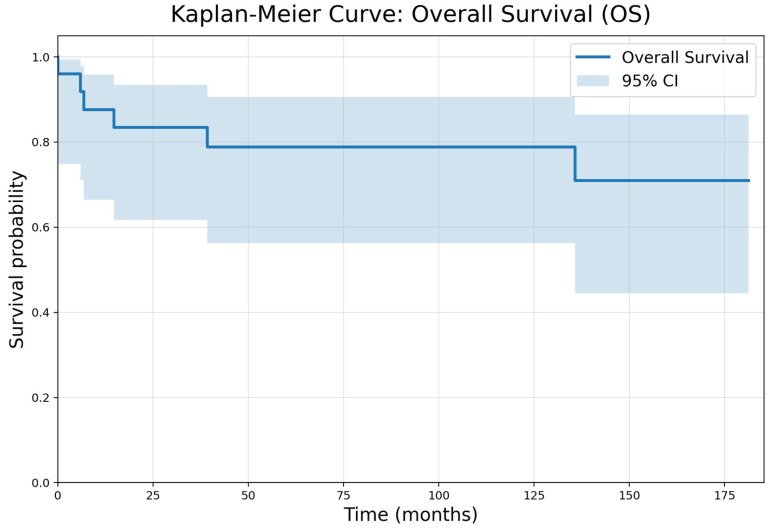
Kaplan–Meier overall survival.

Survival outcomes by histology are summarised in Table 3. Kaplan–Meier curves are shown in Figure 2 and Figure 3. At a median follow-up of 107.9 months (range 4.5–181.3 months), overall survival for the cohort was 76%. Among RCC patients, recurrence occurred in 3/7 patients with four deaths, resulting in an overall survival of 43%. Median follow-up for RCC was 3.6 years (range 1.2–14.5). All CCSK patients were alive at last follow-up with no recurrences, corresponding to an overall survival of 100%. Median follow-up for CCSK was 13.3 years (range 5–15.1). The single patient with anaplastic sarcoma of the kidney remained alive without recurrence at 10.6 years of follow-up. All CMN patients survived, with one patient developing recurrence in both lungs; the median follow-up was 5.45 years (range 0.4–9). Both MRTK patients died during follow-up due to disease progression, corresponding to an overall survival of 0%, with a median follow-up of 0.53 years (range 0.49–0.56). One patient was managed with palliative intent from diagnosis and died five months later, while the second died six months after diagnosis before completing planned treatment. Seven EFS events occurred during follow-up; there were four deaths in the RCC cohort (EFS 42.9%), comprising three relapses and one disease progression, one relapse in the CNM group (EFS 83.3%), and two deaths due to disease progression in patients with MRTK (EFS 0%). No EFS events were observed in patients with CCSK (EFS 100%) or anaplastic sarcoma (EFS 100%).

### 3.7. Recurrence and Salvage Therapy

Tumour recurrence occurred in 4/25 patients (16%). Three recurrences occurred in patients with RCC, all presenting with distant disease involving the liver, lung and bone, respectively. One patient developed recurrence three years after diagnosis and was treated with sorafenib, remaining alive at last follow-up. A second patient experienced distal nodal recurrence in the liver bed five years after diagnosis and underwent surgical excision followed by pazopanib, axitinib and nivolumab, dying one month later. A third patient developed early recurrence within three months of surgery and received palliative pazopanib, dying several weeks later. As mentioned above, one patient with RCC was initially managed as WT and subsequently succumbed to disease progression of their RCC.

One patient with CMN (Cellular histology) developed pulmonary recurrence nine months after diagnosis and was treated with chemotherapy (vincristine, doxorubicin and cyclophosphamide for three cycles, followed by vincristine, actinomycin D and cyclophosphamide for seven cycles), bilateral pulmonary metastasectomy including left lower lobectomy with partial diaphragmatic resection and repair using a patch, and whole-lung radiotherapy (15 Gy in 10 fractions).

### 3.8. Perioperative Complications

Two major perioperative complications were observed in patients with congenital mesoblastic nephroma (CMN): an iatrogenic inferior vena cava injury requiring vascular reconstruction (*n* = 1) and intra-operative tumour rupture complicated by massive haemorrhage necessitating prolonged PICU admission (*n* = 1). Both patients remained alive under ongoing follow-up and, to date, have experienced no long-term complications attributable to surgical morbidity.

### 3.9. Long-Term Toxicities

Patients underwent standard toxicity surveillance where appropriate, including baseline and interval echocardiography and audiology assessment prior to potentially cardiotoxic or ototoxic treatment.

RCC: Late effects included contralateral renal scarring following radiotherapy (*n* = 1). Pazopanib therapy was associated with musculoskeletal toxicity requiring dose reduction in one patient (*n* = 1). A separate patient developed hair depigmentation during treatment (*n* = 1), which later resolved. CCSK: Treatment-related late effects included radiotherapy-associated scoliosis (*n* = 1), anthracycline-related left ventricular systolic dysfunction with dilatation (*n* = 2), unilateral ototoxicity (Brock grade 1) following carboplatin (*n* = 1), and chemotherapy-associated ovarian follicular depletion (*n* = 1). Renal sarcoma: Ifosfamide-related encephalopathy occurred during treatment (*n* = 1), with subsequent delayed puberty (*n* = 1) and renal calculi on follow-up (*n* = 1).

## 4. Discussion

This study represents the first national Irish report describing paediatric NWRTs and provides outcome data within a centralised European service. Overall survival of 76% in our cohort is comparable with previously reported institutional outcomes, although interpretation must consider the higher proportion of aggressive histologies in this series.

Registry analyses have demonstrated marked variability between tumour subtypes, with survival exceeding 95% in CMN but remaining below 20% for metastatic MRTK [4,5]. The presence of Stage IV disease across RCC, MRTK and selected CCSK patients in our cohort mirrors observations from European national datasets, where aggressive histologies frequently present with advanced disease at diagnosis [4,5].

RCC, the most common primary malignancy of the kidney in adults, is rare in children younger than 15 years, accounting for only 2 to 6% of paediatric malignant renal tumours. Translocation-type paediatric RCCs are characterised by specific chromosome translocations involving the transcription factor gene TFE3 located on Xp11.2 or, rarely, TFEB located on 6p2. The most significant difference to adult RCC is the better outcome of paediatric RCC with regional lymph node metastases without distant metastases, with survival rates of over 70% without adjuvant therapy. The majority of paediatric RCCs are localised diseases, which can be cured with surgical tumour resection alone (survival rate over 90%) [4,5]. CCSK is an uncommon renal tumour that comprises 3–5% of all primary renal tumours in children. This tumour is observed most often in children between 2 and 4 years of age and is characterised by a highly malignant potential. A clonal balanced translocation involving t(10;17)(q22;p13) is consistently identified in CCSK. Recurrent internal tandem duplications (ITD) of the X-linked BCL-6 co-repressor (BCOR) gene have been described in CCSK. With current intensive treatment schedules, including radiotherapy and multiagent chemotherapy regimens, outcome has significantly improved (5-year EFS ranging from 65 to 85%, 5-year OS ranging from 75 to 90%) [2,5,6,13]. MRTK is a highly malignant childhood tumour characterised by the loss of INI1 expression in immunostaining, usually sharing biallelic SMARCB1 or SMARCA4 inactivation. MRTK shows an early onset with a median age at diagnosis of 10–18 months, and up to 40%patients have metastasis at diagnosis. Multimodal treatment has been unsatisfactory so far, resulting in 20–40% OS. CMN is a rare tumour that accounts for about 3% of all paediatric renal tumours. However, it is the most common renal neoplasm in the first 3 months of life. Outcomes for patients with CMN are generally excellent when treated with nephrectomy only, with overall survival rates of about 95% [2,6,9,11,18,19,20].

Treatment strategies in NWRTs reflect contemporary SIOP-based risk-adapted management, with neoadjuvant chemotherapy predominantly utilised in CCSK and surgical-first approaches applied to RCC and CMN [2,5,6]. In European cooperative group analyses, CCSK relapse rates of approximately 15% have been reported despite multimodal therapy [21]. The absence of CCSK recurrence in our series may therefore represent improved multidisciplinary coordination or evolving molecular classification rather than a fundamental survival difference.

Compared with the Indian tertiary-centre study, where only a 59% overall survival was reported and neoadjuvant chemotherapy was applied inconsistently across histologies [13], our cohort demonstrates more uniform treatment pathways. Differences may reflect centralised decision-making and access to specialised paediatric oncology expertise rather than intrinsic biological variation.

Population-based registries such as SEER report NWRT incidence rates of approximately 1 per million children annually within an overall paediatric renal tumour incidence of 9–10 per million [3,5,12]. In contrast, institutional cohorts frequently demonstrate enrichment of aggressive subtypes; for example, the Indian single-centre study reported a disproportionately high number of CCSK and MRTK cases relative to national registry data [13]. The comparatively high proportion of Stage IV disease observed in our cohort likely reflects similar referral bias within a national tertiary centre rather than true epidemiological differences.

Overall survival of 76% in this Irish cohort compares favourably with published European experiences. The Dutch national study reported overall survival of approximately 20% for MRTK [5], consistent with the early mortality observed among our metastatic MRTK patients. CMN outcomes remain excellent internationally, with surgery alone achieving survival rates approaching 100% in multiple series, findings mirrored within our cohort [5].

Paediatric RCC outcomes remain heterogeneous across published cohorts. European series have reported recurrence rates ranging from 20 to 30%, particularly in metastatic or high-stage disease [6,8,11]. Recurrence in our study occurred exclusively within the RCC subgroup (3/7 patients), supporting the concept that paediatric RCC behaves as a biologically distinct entity compared with other NWRTs. Our RCC OS rate of 43% is lower than reported in larger contemporary series (e.g., AREN03B2). Notably, 85% of our RCC patients presented with Stage III or IV disease, and 42% had either TFE3 rearrangements or TP53 mutations, which may account for the diminished OS observed in our cohort compared to series with more localised cases and less aggressive tumours. TFE3 rearrangements in RCC commonly result in an unfavourable prognosis, and TP53 mutations are uncommon in RCC but they are strongly associated with poor prognosis, higher tumour grade, and increased risk of metastasis. Differences in relapse patterns compared with earlier European cohorts, where CCSK contributed significantly to late recurrence, may reflect improvements in molecular risk stratification and contemporary chemotherapy protocols [6,8,11,21,22].

A major strength of this study is its extended follow-up duration, with several patients exceeding ten years of surveillance. While many institutional reports are limited by a median follow-up of fewer than five years, restricting the assessment of late relapses [13], our long-term data provide critical insights into durable remission in CMN and the ongoing recurrence risk in paediatric RCC. Nonetheless, the two-decade study period encompasses significant evolution in imaging modalities, staging criteria, and systemic therapies, reflecting broader shifts in paediatric oncology practice over time.

Advances in molecular diagnostics are increasingly influencing risk stratification in NWRTs. Recent studies have identified genetic alterations and epigenetic signatures that may refine prognostication in CCSK and RCC, allowing more tailored treatment intensity and surveillance strategies [2,6,9,11,18]. In parallel, the identification of underlying cancer predisposition syndromes has evolved significantly over the study period, with expanded access to genomic testing improving recognition of patients with inherited risk. Integration of molecular profiling and germline evaluation into routine clinical pathways is therefore likely to become a central component of future international protocols.

Parallel developments in surgical technology are also reshaping paediatric renal tumour management. Robotic-assisted nephrectomy has been reported in selected paediatric oncology centres, offering enhanced visualisation and dexterity in carefully selected cases [23]. Intra-operative indocyanine green (ICG) fluorescence imaging is being explored to improve lymph node mapping and tumour margin identification [24], while patient-specific three-dimensional modelling has demonstrated utility in pre-operative planning for complex renal tumours [25]. Although these technologies were not uniformly used during the historical period of this study, they represent important areas of ongoing innovation that may influence future NWRT treatment strategies.

Key strengths include national case ascertainment within a centralised paediatric oncology service, consistent multidisciplinary decision-making and extended follow-up across multiple histologies. The integration of evolving molecular diagnostics and treatment strategies reflects real-world clinical practice within a European cooperative framework [2,6].

There are a few limitations in our study. The retrospective design and small cohort size limit statistical comparison between histologies. Referral bias within a national tertiary centre may over-represent aggressive tumours relative to population-based registries. Additionally, the extended study period introduces heterogeneity in staging systems, imaging modalities and treatment protocols, complicating direct comparison with contemporary cohorts [3,5]. Analysing a 20-year cohort indeed introduces challenges related to evolving clinical standards, and earlier patients might have been under-staged or categorised differently compared to those diagnosed in the molecular era.

## 5. Conclusions

This national Irish cohort demonstrates that favourable long-term outcomes for children with NWRTs can be achieved within a centralised multidisciplinary system, particularly for CMNs managed surgically. However, aggressive histologies such as MRTK continue to carry poor prognosis, and recurrence remains a major challenge in paediatric RCC. Continued international collaboration, incorporation of molecular diagnostics and adoption of emerging surgical technologies will be essential to further improve outcomes in this rare and heterogeneous group of tumours [2,6,8,9,18,22,26].

## Figures and Tables

**Table 1:** Baseline clinicopathological characteristics by histology.

Histology	*n*	Median Age (Range)	Sex (M/F)	Laterality (R/L)	Stage I	Stage II	Stage III	Stage IV (Metastatic at Diagnosis) *	Nodal Positivity (*n*)	Genetic/Syndromic Associations
RCC	7	7.5 yrs (2–14)	2/5	3/4	1	0	4	2	3	TFE3 (*n* = 2), TP53 (*n* = 1), Mielodysplastic syndrome (*n* = 1)
CCSK	9	2 yrs (7 mo–6 yrs)	2/7	5/4	2	3	3	1	1	BCOR-associated (*n* = 1)
Anaplastic Renal Sarcoma	1	6 yrs	0/1	0/1 1	0	0	0	1	0	DICER1-associated (*n* = 1)
CMN	6	150 days (3–180 d)	3/3	4/2	1	2	3	0	0	None identified
MRTK	2	12.5 mo (6–19 mo)	1/1	1/1	0	0	0	2	2	SMARCB1-associated (*n* = 1)

* Metastatic disease defined by distant spread at presentation.

**Table 2:** Treatment characteristics by histology.

Histology	Upfront Surgery	Neoadjuvant Chemotherapy	Adjuvant Chemotherapy	Adjuvant Radiotherapy	Targeted Therapy (TKI)	Lymph Node Sampling (Patients)
RCC (*n* = 7)	6	1	3	2	4	6
CCSK (*n* = 9)	2	7	9	7	0	9
Anaplastic sarcoma of the kidney (*n* = 1)	1	0	1	1	0	1
CMN (*n* = 6)	6	0	1	0	0	3
MRTK (*n* = 2)	2	0	1	1	0	2

TKI therapy: Targeted therapies included sorafenib, pazopanib, axitinib and nivolumab administered for recurrent or metastatic RCC. Radiotherapy: Primary tumour-site radiotherapy doses varied by histology, with median doses of 10.8 Gy for CCSK, 36 Gy for RCC, 50.4 Gy for anaplastic sarcoma of the kidney and 19.8 Gy for MRTK. Metastatic-site radiotherapy doses are described in Section 3.

**Table 3:** Survival outcomes by histology.

Histology	Recurrence (*n*)	Deaths (*n*)	Overall Survival (%)	Event-Free Survival (%)	Median Follow-Up, Years (Range)
RCC (*n* = 7)	3	4	43%	42.9%	3.3 (1.0–14.5)
CCSK (*n* = 9)	0	0	100%	100%	13.3 (5–15.1)
Anaplastic Renal Sarcoma (*n* = 1)	0	0	100%	100%	10.6 (single case)
CMN (*n* = 6)	1	0	100%	83.3%	4.1 (0.4–9)
MRTK (*n* = 2)	0	2	0%	0%	0.53 (0.49–0.56)

Median follow-up for the overall cohort was 107.9 months (range of 4.5–181.3 months).

## Data Availability

The data presented in this study are available on request from the corresponding author due to privacy and ethical reasons.

## Abbreviations

The following abbreviations are used in this manuscript:

CCSK	Clear Cell Sarcoma of the Kidney
CMN	Congenital Mesoblastic Nephroma
COG	Children’s Oncology Group
EFS	Event-Free Survival
MRTK	Malignant Rhabdoid Tumour of the Kidney
NMRT	Non-Wilms Renal Tumour
OS	Overall Survival
RCC	Renal Cell Carcinoma
SIOP	International Society of Paediatric Oncology
WT	Wilms Tumour
